# IFNAR2 p.F8S Variant Associates with Severe COVID-19 and Adaptive Immune Cell Activation Modulation

**DOI:** 10.3390/ijms27020992

**Published:** 2026-01-19

**Authors:** Francesco Malvestiti, Angela Lombardi, Francesco Gentile, Veronica Torcianti, Elena Trombetta, Alessandro Cherubini, Giuseppe Lamorte, Sara Colonia Uceda Renteria, Daniele Marchelli, Lorenzo Rosso, Alessandra Bandera, Flora Peyvandi, Francesco Blasi, Giacomo Grasselli, Laura Porretti, Saleh Alqahtani, Daniele Prati, Roberta Gualtierotti, Blagoje Soskic, Valentina Vaira, Luisa Ronzoni, Luca Valenti

**Affiliations:** 1 Department of Pathophysiology and Transplantation, Università degli Studi di Milano, 20122 Milan, Italy; francesco.malvestiti@unimi.it (F.M.); daniele.marchelli@unimi.it (D.M.); lorenzo.rosso@policlinico.mi.it (L.R.); flora.peyvandi@unimi.it (F.P.); francesco.blasi@policlinico.mi.it (F.B.); giacomo.grasselli@policlinico.mi.it (G.G.); roberta.gualtierotti@unimi.it (R.G.); valentina.vaira@unimi.it (V.V.); 2 Precision Medicine Lab, Transfusion Medicine and Hematology, Biological Resource Centre, Fondazione IRCCS Ca’ Granda Ospedale Maggiore Policlinico, 20122 Milan, Italy; angela.lombardi@policlinico.mi.it (A.L.); veronica.torcianti@policlinico.mi.it (V.T.); alessandro.cherubini@policlinico.mi.it (A.C.); giuseppe.lamorte@policlinico.mi.it (G.L.); daniele.prati@policlinico.mi.it (D.P.); luisa.ronzoni@policlinico.mi.it (L.R.); 3 Division of Pathology, Fondazione IRCCS Ca’ Granda Ospedale Maggiore Policlinico, 20122 Milan, Italy; francesco.gentile@meduniwien.ac.at; 4 Flow Cytometry Laboratory, Clinical Pathology, Fondazione IRCCS Ca’ Granda Ospedale Maggiore Policlinico, 20122 Milan, Italy; elena.trombetta@policlinico.mi.it (E.T.); laura.porretti@policlinico.mi.it (L.P.); 5 Virology Unit, Fondazione IRCCS Ca’ Granda Ospedale Maggiore Policlinico, 20122 Milan, Italy; sara.ucedarenteria@policlinico.mi.it; 6 Thoracic Surgery and Lung Transplantation Unit, Fondazione IRCCS Ca’ Granda Ospedale Maggiore Policlinico, 20122 Milan, Italy; 7 Infectious Diseases Unit, Fondazione IRCCS Ca’ Granda Ospedale Maggiore Policlinico, 20122 Milan, Italy; alessandra.bandera@policlinico.mi.it; 8 SC Medicine-Haemostasis and Thrombosis, Fondazione IRCCS Ca’ Granda Ospedale Maggiore Policlinico, 20122 Milan, Italy; 9 Respiratory Unit and Cystic Fibrosis Center, Fondazione IRCCS Ca’ Granda Ospedale Maggiore Policlinico, 20122 Milan, Italy; 10 Department of Anesthesia, Critical Care and Emergency, Foundation IRCCS Ca’ Granda Maggiore Policlinico Hospital, 20122 Milan, Italy; 11 Liver, Digestive, and Lifestyle Health Research Section, and Organ Transplant Center of Excellence, King Faisal Specialist Hospital & Research Center, Riyadh 11211, Saudi Arabia; salqaht1@jhmi.edu; 12 Division of Gastroenterology and Hepatology, Weill Cornell Medicine, New York, NY 10065, USA; 13 Human Technopole, Viale Rita Levi-Montalcini 1, 20157 Milan, Italy; blagoje.soskic@fht.org

**Keywords:** immunity, COVID-19, SARS-CoV-2, IFNAR2, dendritic cells

## Abstract

Severe acute respiratory syndrome coronavirus 2 (SARS-CoV-2) infection has a wide range of clinical manifestations modulated by genetic factors. The aim of this study was to identify genetic determinants of severe COVID-19 affecting protein sequence to gain insight into disease pathogenesis. Variants prioritized in two patients requiring lung transplant were tested in the Milan FOGS cohort (487/869 cases/controls), highlighting an independent association between the p.F8S low-frequency variant of interferon alpha receptor 2 gene (*IFNAR2*) and severe disease (OR = 1.73 [1.24–2.42], *p* = 0.001), replicated in the COVID-19 Host Genetics Initiative cohort (26,167/2,061,934 cases/controls). In the FOGS cohort, the p.F8S variant was linked to higher circulating IL-6 levels. In keeping, bulk transcriptomic analysis in PBMCs at the peak of infection (*n* = 57) showed that carriers of the p.F8S variant had upregulation of immune signaling and pathogens response (*p* < 0.05). Functional flow cytometry experiments in healthy donors (*n* = 12) revealed that membrane IFNAR2 protein expression was reduced in B lymphocytes, but higher in dendritic cells (*p* < 0.05). Finally, by interrogating a public scRNAseq resource of PBMC of people with COVID-19, we showed that p.F8S carriers had upregulation of immune pathways specifically in dendritic cells (*p* < 0.05). These results suggest that the p.F8S variant may influence COVID-19 severity by enhancing adaptive immune response, thereby favoring inflammation.

## 1. Introduction

Coronavirus disease 2019 (COVID-19) is caused by Severe Acute Respiratory Syndrome Coronavirus 2 (SARS-CoV-2) infection [1]. The clinical manifestations of SARS-CoV-2 infection encompass a spectrum of scenarios, from the most common asymptomatic or mildly symptomatic condition [2] to severe Coronavirus disease 2019 (COVID-19) [1,3]. The pathogenesis of COVID-19 and the basis of its clinical variability are not yet completely clear. Higher susceptibility to infection in the lower respiratory tract, a cytokine storm with sustained inflammatory response, endothelial perturbation and activation of the coagulation cascade are key pathogenic steps [4,5,6,7]. Genetic factors contribute to determining the individual predisposition to severe COVID-19, particularly in young adults [8,9,10]. Despite the efforts of several authors and international consortia such as the COVID-19 Host Genetics Initiative (HGI) [11], which allowed the identification of the major genetic determinants of disease susceptibility and severity, the common variants highlighted until now explain only a limited proportion of the COVID-19 variability [10,12]. Further investigations are therefore required to shed light on the genetic mechanisms underlying COVID-19 manifestations.

To identify new genetic determinants and contribute to clarifying the mechanisms of severe COVID-19, we focused on protein coding variants with a high likelihood of modifying protein function, enriched in patients with severe disease. As a prioritization strategy, we analyzed whole exome sequencing (WES) data of two patients who required lung transplantation due to irreversible respiratory failure [13]. Among candidates, we identified and validated in a global cohort a low-frequency variant affecting *IFNAR2* (rs2229207 T>C missense variant, encoding for the p.F8S substitution). The *IFNAR2* gene is located at chromosome 21 at the same locus as *IL10RB*, and it has translational relevance as it encodes for a receptor that is targeted by IFNα. Remarkably, the *IFNAR2* locus independently emerged from previous genome-wide association studies (GWAS) as one of the main genetic determinants of COVID-19 severity. In particular, the non-coding rs2236757 variant at the *IFNAR2* locus was associated with severe COVID-19, while the intronic rs13050728 with protection against critical illness and hospitalization [8,14]. Although rs2236757 and rs13050728 are common variants with high allele frequencies in population databases such as GnomAD, they have been consistently associated with COVID-19 severity in multiple GWAS, reflecting modest effect sizes typical of common risk alleles in complex diseases rather than high-penetrance pathogenic variants. In addition, the risk of severe COVID-19 was inversely associated with *IFNAR2* mRNA expression [8]. However, the mechanisms leading to dysfunctional interferon (IFN)-dependent immune response to SARS-CoV-2 remain unclear [8,14,15,16].

Here, through an integrative approach including clinical and biomarker data evaluation, transcriptomic analysis of immune cells isolated from SARS-CoV-2 infected patients and in vitro functional characterization studies on peripheral blood mononuclear cells (PBMCs) of healthy subjects, we aimed to describe the immune pathways linking *IFNAR2* with severe COVID-19.

## 2. Results

### 2.1. IFNAR2 rs2229207 T>C p.F8S Variant Associated with Severe COVID-19

**Figure 1:**
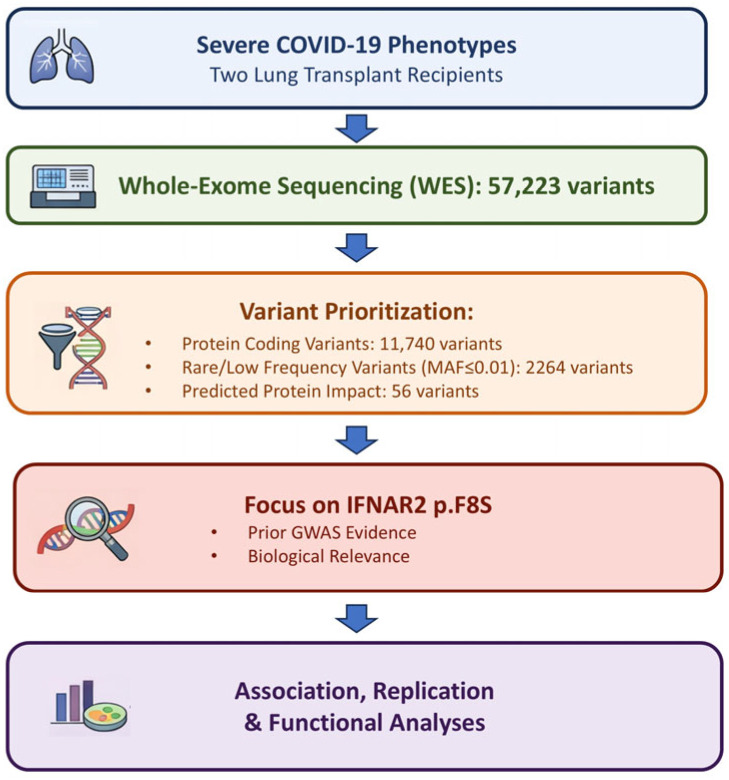
Overview of the variant prioritization pipeline. Schematic representation of the analytical workflow used to identify the *IFNAR2* p.F8S variant, including extreme-phenotype selection, whole-exome sequencing, variant filtering based on allele frequency and predicted functional impact, candidate prioritization, and downstream association and functional analyses.

First, to prioritize a set of protein coding genetic variants that may predispose to severe respiratory failure in patients with COVID-19, we performed whole exome sequencing (WES) of two previously characterized young Italian male patients who underwent lung transplantation due to irreversible respiratory failure [13]. We identified 57,223 variants, of which 11,740 are protein coding or splicing sites, and applied a prioritization pipeline based on frequency, predicted impact and functional annotation in clinical databases [17,18]. Briefly, we selected coding and splicing variants based on their allele frequency, focusing the attention on those with minor allele frequency (MAF) < 0.1 in GnomAD non-Finnish European (NFE), highlighting 2264 variants. An overview of the variant prioritization strategy is shown in Figure 1.

**Figure 2:**
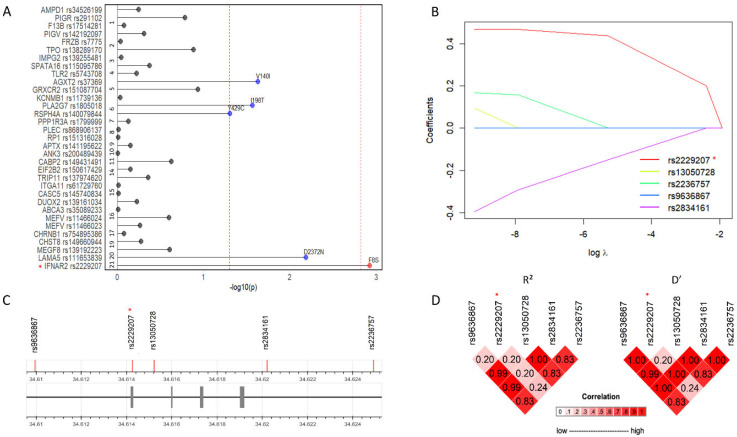
Association analysis. (**A**): *IFNAR2* rs2229207 T>C encoding the p.F8S variant associates with severe COVID-19 within the FOGS cohort (padj = 0.047, OR (95% C.I.) = 1.73 (1.24–2.42)) (487 patients vs. 869 blood donors). (**B**): Conditional analysis of the top significant *IFNAR2* variants according to the literature showed rs2229207 p.F8S as the variant with the higher estimate (β = 0.47) associated with the trait. (**C**): Schematic representation of the *IFNAR2* variants considered in the LD and Conditional analyses. (**D**): Linkage disequilibrium analysis suggests a non-random association between rs2229207 T>C variant and COVID-19 outcome. (*) highlights the variant identified during the prioritization phase.

Then, we selected pathogenic variants based on ClinVar, prioritizing a set of 56 variants distributed in 55 genes (Table S1). Next, we tested these variants for the association with severe COVID-19 in a case–control cohort of 487 patients and 869 healthy blood donors with available genotyping, geographically and temporally matched to the cases during the first wave of infection (2020, Milan Fondazione Genomic Study [FOGS] cohort) [10]. As rare variants were not typed nor could be reliably imputed, in this cohort we were able to identify only 42 out of the 56 variants, and among these, 9 were monomorphic and were then excluded from the analysis. By multivariable logistic regression, after applying frequency and functional impact filters, five coding variants meeting these criteria were retained for further analysis and as a result nominally associated with severe COVID-19 (*AGXT2* rs37369, *PLA2G7* rs1805018, *RSPH4A* rs140079844, *LAMA5* rs111653839, *IFNAR2* rs2229207; Table S1). After Bonferroni correction, only *IFNAR2* rs2229207 T>C missense variant, encoding the p.F8S substitution, remained associated with the risk of severe COVID-19 (Figure 2A, *p* = 0.001, adjusted *p* = 0.047, OR = 1.73, 95% C.I. = 1.24–2.42, MAF [NFE] = 0.08). The association remained significant after adjustment for demographic factors and for the main genetic loci for susceptibility to and severity of COVID-19 at *ABO* and *LZFTL1*, respectively [10] (Table 1, *p* = 0.001).

Since previous GWAS identified variants at the *IFNAR2* locus as risk factors for COVID-19 severity [8,10,14,19], to rule out that the association of *IFNAR2* p.F8S variant with severe COVID-19 was due to linkage with risk alleles within the 21q22.11 locus, we performed a linkage disequilibrium (LD) analysis in the FOGS cohort among the polymorphisms in the *IFNAR2* gene associated with COVID-19 susceptibility (Figure 2C,D). All the SNPs we tested showed a low correlation with *IFNAR2* p.F8S variant (range R^2^ = 0.20–0.24, range D’ = 0.20–0.24), indicating limited correlation between *IFNAR2* p.F8S and previously reported variants at the locus, and suggesting that the observed association is unlikely to be explained by strong linkage disequilibrium.

We therefore tested whether the association of *IFNAR2* p.F8S with severe COVID-19 was independent of top hit variants reported in previous GWAS (i.e., rs13050728 [14], rs9636867 and rs2236757 [8]) (Table 1). Remarkably, correction for these variants did not appreciably attenuate the association of *IFNAR2* p.F8S with severe phenotype (Table 1, *p* = 0.017, OR = 1.60, 95% C.I. = 1.09–2.34), and *IFNAR2* p.F8S remained the only suggestive predictor showing a distinct association with severe COVID-19 at this locus also when other variants were introduced separately in the model (Table S2).

Next, to validate the association of *IFNAR2* p.F8S with severe COVID-19 phenotypes, such as hospitalization due to COVID-19, we interrogated the publicly available HGI [11] meta-analysis results (*n* = 26,167 cases and *n* = 2061,934 controls). Consistent with previous results, in Europeans the p.F8S variant showed an association with hospitalization due to COVID-19 (Table S3, *p* < 0.001, OR = 1.11, 95% C.I. = 1.07–1.14). To confirm the independence of the association of the p.F8S variant from other hits at the *IFNAR2* locus, we performed, in the FOGS and HGI cohorts, summary statistics, a conditional analysis employing the GCTA-SOJO software (version 2.0). The p.F8S missense variant retained the larger estimate of increased risk (Table S3, β = 0.47) among the variants considered (i.e., rs13050728, rs9636867, rs2236757 and rs2834161; Figure 2B).

Taken together, these results suggest that *IFNAR2* p.F8S may represent a genetic modifier contributing to the increased risk of severe COVID-19 linked with the *IFNAR2* locus.

### 2.2. Impact of IFNAR2 rs2229207 T>C p.F8S on Clinical and Virological Features

To elucidate the mechanism leading to an enrichment of the p.F8S variant in patients with severe COVID-19, we examined its association with biomarkers and clinical outcomes in the FOGS cohort.

To this end, we analyzed the impact of *IFNAR2* p.F8S on circulating markers of inflammation at the time of hospitalization, i.e., C-reactive protein (CRP), ferritin, neutrophil-to-lymphocyte ratio (NLR) and interleukin 6 (IL-6). At multivariable linear regression, adjusted for age, biological treatment and steroid treatment, *IFNAR2* p.F8S was associated with higher circulating IL-6 (*n* = 112, *p* = 0.046, OR = 1.61, 95% C.I. = 1.01–2.58; Table S4). On the other hand, the p.F8S variant was not significantly associated with D-Dimer, SARS-CoV-2 antibodies or SARS-CoV-2 viral load (Figure S1; Supplementary Results).

At multivariable logistic regression adjusted for age, gender, the non-coding *IFNAR2* rs13050728 and steroid treatment, the p.F8S variant was not associated with in-hospital mortality (*p* = 0.65, Table S4). However, among hospitalized patients, carriage of the p.F8S variant was inversely associated with the need of intensive respiratory support during hospitalization (Tables S4 and S5, *p* = 0.021, OR = 0.86, 95% C.I. = 0.76–0.98), with lower frequency of intensive respiratory support among carriers. This finding reflects a conditional association within the hospitalized cohort and should not be interpreted as a protective effect against severe COVID-19. This analysis was adjusted for age, gender, hydroxychloroquine, steroids and low-molecular weight heparin treatments, *IFNAR2* rs13050728, *ABO* and *LZFTL1* top hit variants from previous GWAS.

These results suggest that while the carriage of *IFNAR2* p.F8S predisposes to severe COVID-19 leading to hospitalization, the mechanism does not seem to be related to increased vulnerability to progressive lung damage, coagulopathy, nor to impaired control of viral replication, but it may encompass a pro-inflammatory effect linked to upregulation of IL-6 secretion.

### 2.3. Impact of IFNAR2 p.F8S on PBMCs Transcriptomic Profiles in Patients with Severe COVID-19

As *IFNAR2* is expressed on circulating immune cells, to clarify the mechanism by which *IFNAR2* p.F8S predisposes individuals to severe COVID-19, we next investigated the impact of the p.F8S variant on the immune cell transcriptome during SARS-CoV-2 infection in 57 patients with severe disease from the FOGS cohort (of whom *n* = 8 were carriers of p.F8S—heterozygous, *n* = 7; homozygous, *n* = 1), whose PBMCs were collected at the peak of infection during hospitalization.

**Figure 3:**
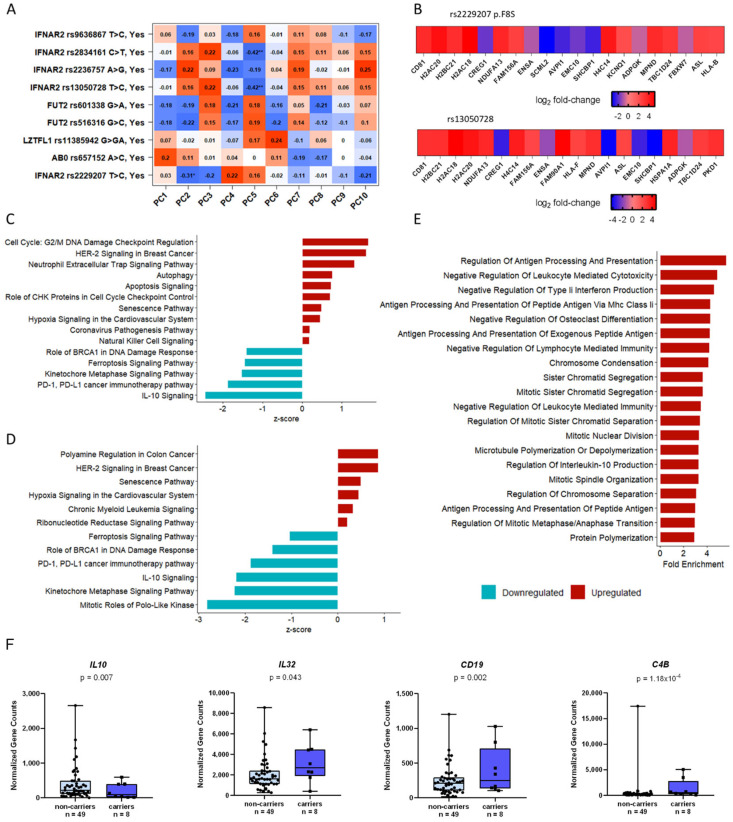
Transcriptomic analysis of PBMCs of fifty-seven COVID-19 patients at the peak of the infection. (**A**): Principal component analysis (PCA). The *IFNAR2* p.F8S variant represents the most relevant genetic determinants of PBMCs transcriptome in SARS-CoV-2 infected patients as it associates with PC2. * and ** refer to the significance added by default by DESeq2 version 1.44.0. (**B**): Top 20 differential expressed genes (DEGs) in *IFNAR2* p.F8S carriers vs. non-carriers (upper) and top 20 DEGs in the carriers of p.F8S carriers, when the rs13050728 intron variant was accounted for in the analysis (lower). (**C**): Enriched pathways in *IFNAR2* p.F8S carriers vs. non-carriers. (**D**): Enriched pathways in *IFNAR2* p.F8S carriers vs. non-carriers accounting for the rs13050728 intron variant genotype carriage contribution in gene expression of COVID-19 patient’s PBMCs at the peak of the infection. (**E**): Overrepresentation analysis (ORA) in p.F8S carriers vs. non-carriers. Differential expression analysis highlighted a set of 1729 DEGs, 1171 downregulated and 558 upregulated. Genes showing a log_2_ fold change higher than 1 or less than −1 were considered in the ORA; gene ontology (GO) terms which were overrepresented among the DEGs of patients carrying the p.F8S variant indicated an enrichment in biological processes linked to leukocytes function such as the regulation of antigen presentation and processing via class II MHC, cell mediated cytotoxicity and the regulation of type II IFNs and IL-10 production. (**F**): Marker genes identified in the differential expression analysis. IL-10 showed to be underregulated in the carriers of the p.F8S. Conversely, *IL-32*, *CD19* and *C4B* were overexpressed in carriers of the same variant.

First, principal component analysis (PCA) was performed to pinpoint the most important determinants of transcriptome variability (Figure 3A). PC2 was associated with carriage of *IFNAR2* rs2229207 p.F8S (β = −0.31, *p* < 0.05), supporting a functional impact on circulating immune cells (Figure 3A). Notably, no correlation was found between the transcriptome variability and top variants associated with COVID-19 phenotype in previous GWAS, namely *LZTFL1* rs11385942 G>GA, *ABO* rs657152 A>C, *FUT2* rs516316 G>C nor *FUT2* rs601338 G>A. On the other hand, a smaller fraction of the variability was explained by the *IFNAR2* rs13050728 and rs2834161 non-coding variants (PC5; β = −0.42, *p* < 0.01) (Figure 3A).

We then investigated the impact of p.F8S carriage on immune cell bulk transcriptome by differential expression analysis, adjusting for age, gender, lymphocytes/monocytes ratio and the top hits highlighted in previous GWAS [10,14,19]. This analysis revealed a set of 1729 differentially expressed genes in patients carrying the p.F8S (Table S6; 1171 genes were downregulated and 558 upregulated).

Although no significant difference was found in the expression of *IFNAR2* (*p* = 0.82; Figure S2A) and *IFNAR1* (*p* = 0.79; Figure S2B) genes in association with p.F8S, we detected a significantly lower *IFNAR2*/*IFNAR1* expression ratio (*p* = 0.02; Figure S2C) in carriers of the variant.

The top 20 differentially expressed genes (DEGs) in the carriers of the p.F8S, including after adjustment for the rs13050728 intronic variant (GWAS top hit) are shown in Figure 3B. The most significantly upregulated gene in p.F8S carriers was *CD81* (log_2_-fold change = 3.13, *p* = 1.06 × 10^−10^, FDR = 7.87 × 10^−7^), encoding for a structural component of tetraspanin-enriched microdomains (TERMs) involved in adhesion-dependent immune cell migration [20] and coronavirus infections, by facilitating viral entry and fusion with host cell membrane expressed on the membrane of B cells, T cells and dendritic cells [20]. In addition, *CD19*, which encodes for a structural protein interacting with CD81 protein upon immune activation of B cells, was also upregulated (log_2_-fold change = 1.63, *p* = 4.85 × 10^−5^, FDR = 0.002) (Figure 3C).

Next, we employed Ingenuity Pathway Analysis (IPA) to determine the specific contribution of the p.F8S variant on biological processes at the peak of SARS-CoV-2 infection, also additionally adjusting the analysis for *IFNAR2* rs13050728 intronic variant (Figure 2D). The p.F8S variant was linked to overexpression of cell cycle regulation, genes linked to COVID-19 pathogenesis and several pathways involving immune cell response to pathogens (Figure 3C). Furthermore, pathways related to cell senescence and programmed cell death (ferroptosis) were upregulated, while *PD-1*/*PD-L1* immunotherapy and *IL-10* signaling pathways were downregulated, suggesting an alteration of T cell exhaustion-related regulatory mechanisms in response to SARS-CoV-2 infection (Figure 2D). Overrepresentation analysis (ORA) revealed enrichment in biological processes linked to leukocytes’ function such as the regulation of antigen presentation and processing via class II major histocompatibility complex (MHC), cell mediated cytotoxicity and regulation of type II IFNs and IL-10 production (Figure 3E), whose expression resulted altered in p.F8S carriers vs non-carriers.

All in all, results suggest that the carriage of *IFNAR2* p.F8S is associated with upregulation of a transcriptional program sustaining immune response following SARS-CoV-infection.

### 2.4. Impact of IFNAR2 p.F8S on IFN-α Signaling in PBMCs

Next, to gain further insight into the mechanism whereby the p.F8S variant affects immune cells, we investigated the impact on the transcriptional profiles of PBMCs isolated from healthy blood donors wild-type (*n* = 4), heterozygous (*n* = 4) and homozygous (*n* = 4) for the *IFNAR2* p.F8S variant, in resting conditions or exposed to IFN-α. We evaluated the relative expression of *IFNAR2*, *STAT2* and *ISG15* genes which represent the receptor, the trans-activator and the effector during SARS-CoV-2 infection, respectively. In keeping with results obtained during COVID-19, the p.F8S variant was associated with lower *IFNAR2* mRNA levels, both at baseline and following IFN-α exposure (β = −0.502, *p* = 0.010; Table S7 and Figure S3A). Interestingly, although a general increase in the transcription of the *STAT2* gene was detected following IFN-α, lower *STAT2* expression was observed in carriers of the p.F8S variant (β = −0.541, *p* = 0.028; Figure S3B and Table S7). Conversely, the expression of *ISG15* was not affected by the p.F8S variant, although it was induced upon IFN-α exposure (Figure S3C).

In the same experiment, we also compared the effect on the same outcomes of the *IFNAR2* rs13050728 intronic variant linked with protection against COVID-19 (wild-type, *n* = 6; heterozygous, *n* = 3; homozygous, *n* = 3). We found that the rs13050728 variant was associated with a specular increase in *IFNAR2* expression (β = 0.396, *p* = 0.043), but did not impact either *STAT2* nor on *ISG15* expression (*p* = 0.583 and *p* = 0.428, respectively; Table S7).

These results suggest that the p.F8S variant impacts *IFNAR2* and *STAT2* in vitro gene expression in healthy PBMCs even in the absence of IFN-α stimulation. In addition, the protective rs13050728 exerts a specular but less marked effect on *IFNAR2* expression than the p.F8S variant.

### 2.5. IFNAR2 p.F8S Is Linked with Altered Abundance of IFNAR2^+^ Circulating Immune Cell Populations and Cell Surface IFNAR2 Expression

We next examined the impact of the p.F8S variant on IFNAR2 protein expression in circulating immune cells by flow cytometry, to identify the main immune sub-populations expressing this receptor, namely T lymphocytes (CD4^+^ and CD8^+^), B lymphocytes, natural killer (NK) lymphocytes, monocytes, neutrophils, and dendritic cells (monocytoid, mDC, and plasmocytoid, pDC [21]). We quantified both the mean fluorescence intensity (MFI), reflecting the expression level, of IFNAR2 protein on the cell surface and the absolute number of IFNAR2^+^ cells in 8 healthy individuals stratified by carriage of IFNAR2 p.F8S variant (*n* = 5 carriers vs. *n* = 3 non-carriers). Due to a partial LD, subjects negative for the p.F8S variant (*n* = 3) were homozygous for the rs13050728 intronic variant and vice versa (Table S8).

**Figure 4:**
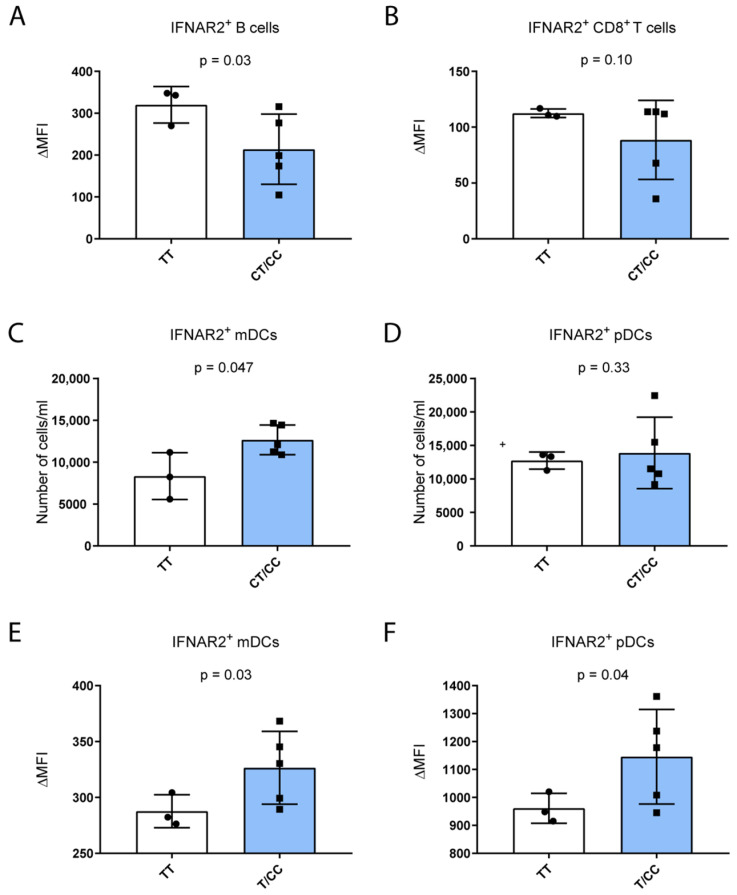
Association study of heathy PBMC composition and IFNAR2 protein expression on the cell surface of PBMC sub-populations (*n* = 8). (**A**–**D**): The absolute number of IFNAR2^+^ mDCs increases according to the carriage of the p.F8S variant while the IFNAR2^+^ fraction of B cells was shown to decrease according to p.F8S carriage; the IFNAR2^+^ cells within the CD8^+^ T cell fraction indeed is inversely associated with the carriage of the variant. (**E**,**F**): In addition, the increase in the mean fluorescent intensity of the signal in IFNAR2^+^ fraction of mDCs and pDCs reflects an increase in protein expression, while a negative association was found for the IFNAR2 expression on the surface of CD8^+^ T and B cells and the p.F8S carriage. Data are shown for individual samples to illustrate variability; statistical significance should be interpreted with caution due to the limited number of WT controls.

In keeping with bulk RNAseq data, carriage of the p.F8S variant was associated with reduced receptor expression in IFNAR2^+^ B lymphocytes (Figure 4A, *p* = 0.03), the largest population expressing the protein. In CD8^+^ (Figure 4B) and CD4^+^ T cells and NK cells (Table S9) we did not observe any difference in IFNAR2 expression according to variant carriage.

Notably, p.F8S variant carriers showed a higher number of IFNAR2^+^ mDCs (*p* = 0.047; Figure 4C), whereas no differences were found within the IFNAR2^+^ pDCs sub-population (*p* = 0.33; Figure 4D) between the two groups. Consistently with the increased abundance of IFNAR2^+^ mDCs, carriers presented a higher mDC surface expression of the receptor vs. non-carriers (*p* = 0.03; Figure 4E). Similarly, in IFNAR2^+^ pDCs, the expression of the receptor was also significantly increased in the p.F8S carriers vs. non-carriers (*p* = 0.04; Figure 4F).

Given the limited number of WT controls and the substantial inter-individual variability observed among p.F8S carriers, the results shown in Figure 4 should be interpreted as exploratory and descriptive. However, taken together, these results suggest a potential role for the IFNAR2 p.F8S variant in promoting inflammation though the upregulation of IFNAR2 protein activity on the cell surface within the main DC sub-populations.

### 2.6. Enhanced Immune Activation of DCs in p.F8S Carriers

Given this scenario, to further investigate the hypothesis that the p.F8S variant may alter immune response and promote inflammation in carriers in specific immune population subsets, we analyzed publicly available single-cell RNA sequencing (scRNA-Seq) data of eight SARS-CoV-2-infected patients from two different studies [22,23].

We called the p.F8S genotype exploiting Monopogen [24] software (v1.6.0) (wild-type, *n* = 2; heterozygous, *n* = 3; homozygous, *n* = 3) and then we performed gene set enrichment analysis (GSEA) considering significant DEGs (*p* < 0.05) for specific immune cell sub-populations in carriers vs. non-carriers of the p.F8S variant.

**Figure 5:**
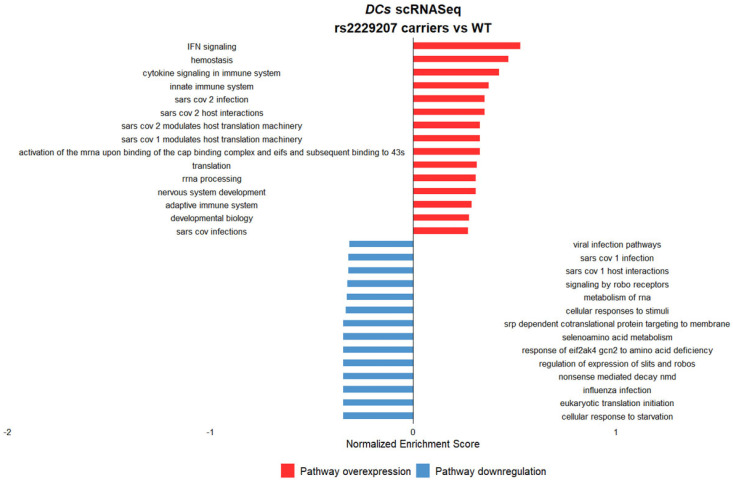
Gene set enrichment analysis of differentially expressed genes in DCs stratified for the carriage of the p.F8S variant (carriers = 6 vs. non-carriers = 2). The carriers of the variant showed an enhanced expression of IFN-related and inflammatory pathways linked to cytokine storm and immune system. In addition, we observed an upregulation of SARS-CoV-2-related pathways in the carriers vs. non-carriers.

Single-cell analysis revealed cell-type–specific transcriptional patterns associated with p.F8S carrier status. We specifically identified in DC an upregulation of pathways related to innate and adaptive immune system, inflammatory pathways such as IFN and cytokine signaling, and pathways involved in SARS-CoV-2 infection and modulation in carriers of the risk p.F8S variant (Figure 5).

Altogether, these data are consistent with a specific impact of the p.F8S variant on immune activation in DCs, likely through increased IFNAR2 activity on the cell surface.

## 3. Discussion

In this study, we first identified the low-frequency p.F8S missense variant at the *IFNAR2* gene locus as an independent genetic marker associated with COVID-19 severity.

Previous genome-wide association studies have identified multiple human genetic loci associated with COVID-19 susceptibility and severity. The most consistently replicated signals include the 3p21.31 locus (encompassing *SLC6A20*, *LZTFL1*, and chemokine receptor genes), the *ABO* blood group locus, and loci near *OAS* gene clusters, *TYK2*, and *DPP9*, which implicate innate immune and inflammatory pathways. Remarkably, the *IFNAR2* region on chromosome 21q22.11 has been repeatedly associated with severe COVID-19 in multiple GWAS and meta-analyses, and integrative genetics studies suggest that variation at this locus may influence disease risk via effects on interferon signaling and immune cell gene expression. These findings underscore the polygenic nature of COVID-19 outcomes and the involvement of interferon and innate immune pathways in severe disease [10,11,12,14].

By analyzing local and global cohorts, we showed that an additional, low frequency *IFNAR2* variant, namely p.F8S, associates with an increased risk of severe COVID-19 requiring hospitalization. The p.F8S variant displayed a low linkage with the leading *IFNAR2* gene variants associated with severe COVID-19 susceptibility in previous GWAS, and the association with severe disease outcomes persisted after extensive correction for multiple genetic and clinical risk factors [4,5,6,10]. This scenario was also supported by the results of the conditional analysis, showing that p.F8S has the largest effect size on the risk of severe COVID-19 among *IFNAR2* variants in the global HGI cohort. The attenuation of effect size observed in the COVID-19 Host Genetics Initiative in comparison to the FOGS cohort is consistent with replication of a low-frequency variant in a population-based meta-analysis and does not undermine the robustness of the association, which is supported by consistent directionality across datasets of very different design and scale. Although p.F8S shows low linkage disequilibrium with previously reported *IFNAR2* variants, formal demonstration of signal independence would require conditional analyses in large cohorts with individual-level data; therefore, independence should be regarded as suggestive rather than definitive. Because variant prioritization was applied, statistical significance should be interpreted conservatively and in conjunction with independent replication. However, this hypothesis also has a biological plausibility, as the *IFNAR2* p.F8S substitution maps to the extracellular N-terminal region of the receptor, which is involved in ligand binding and receptor complex formation. Although no direct structural or functional assays were performed in this study, alterations at this position could plausibly affect receptor stability, surface expression, or ligand interaction efficiency. These structural considerations remain speculative and warrant future experimental investigation.

By exploiting the local database with detailed individual clinical data, we were able to show that the carriage of the p.F8S variant did not render patients sicker due to more severe respiratory involvement. Conversely, the p.F8S variant was associated with more severe systemic inflammation, as estimated by the higher circulating IL-6 levels. Therefore, markers of systemic inflammation were observed in the absence of measurable differences in viral load or canonical antiviral interferon responses.

To gain insight into this double-edged impact of *IFNAR2* variation on COVID-19, we examined transcriptomic data of PBMCs at the peak of infection at the time of hospitalization and consistently with biomarker data, we observed that the p.F8S variant was associated with enhanced activation of immune pathways and anti-viral responses. The variant may contribute to an immune cell shift towards the upregulation of genes able to trigger systemic inflammation. Interestingly, the most upregulated gene, namely *CD81*, is specifically required for the formation of lamellipodia in migrating DCs [25], suggesting an active role for DC activation, maturation, migration, and survival related to type I IFN-mediated response in the activation of immune cells in COVID-19.

Importantly, we observed that these biological alterations were paralleled by a reduction in the *IFNAR2*/*IFNAR1* expression ratio in carriers of the p.F8S variant. In keeping, we also observed that the p.F8S variant was associated with trends toward reduced *IFNAR2* expression and altered *STAT2* levels, irrespective of IFNα exposure, when carriers were analyzed collectively in total PBMCs of healthy individuals. However, supplementary analyses indicate substantial heterogeneity, with heterozygous carriers frequently exhibiting values closer to WT and homozygous carriers showing more pronounced effects, suggesting a possible dose-dependent but variable influence of the variant. These findings underscore that the molecular consequences of p.F8S are not uniform across carriers and should be interpreted as modest and context-dependent.

Additionally, the variant was associated with reduced IFNAR2 protein expression on the surface of B cells, the largest pool of positive cells in the circulation. Therefore, we hypothesize that the shift towards an altered immune activation may be accounted for by the modulation of IFNAR2 activity on the cell surface of specific immune cells. In particular, we observed that in DCs that showed the higher intensity of membrane protein expression, the p.F8S variant was associated with an increase in the absolute number of circulating IFNAR2^+^ mDCs and an enhanced expression of the receptor in both mDCs and pDCs. Although DCs account for a small proportion of the total PBMC ensemble, they can respond to type I IFN also by generating an autocrine circuit [26] promoting a global shift towards a pro-inflammatory state. The differential association of p.F8S with IFNAR2 expression across immune cell subsets highlights the context-dependent nature of interferon receptor regulation. IFNAR2 expression and signaling are known to vary substantially between lymphoid and myeloid lineages, and changes in receptor abundance do not necessarily translate into proportional downstream signaling. In this context, the observed increase in IFNAR2 expression in dendritic cells, contrasted with reduced expression in bulk PBMCs and B cells, should be interpreted as cell-type-specific correlates rather than evidence of globally enhanced interferon activity.

Consistent with this interpretation, p.F8S carrier status was not associated with increased antiviral responses, as reflected by unchanged viral load, seroconversion rates, and *ISG15* expression, and by reduced *STAT2* levels. Together, these findings argue against a model of enhanced canonical interferon signaling. An alternative explanation is that subtle, cell-type-specific alterations in IFNAR2 expression may influence immune cell activation thresholds or inflammatory tone without measurably affecting viral control. However, direct functional studies will be required to test this hypothesis. Additionally, the structural modeling of the p.F8S substitution within the IFNAR2 receptor may help elucidate potential effects on receptor stability or signaling and represents an important avenue for future investigation. Functional analyses were conducted in relatively small cohorts, limiting statistical power and precluding definitive mechanistic inference; these experiments should therefore be regarded as exploratory and hypothesis-generating.

Taken together, these findings suggest that the functional impact of the p.F8S variant may be dependent on the immune cell type, with important implications concerning the employment of interferons for the treatment of coronavirus infections. Particularly, in this study we underlined the need for a better stratification of patients from a genetic point of view in the context of personalized medicine in order to modulate the IFNAR2/1-mediated pathways with the final aim to not trigger, or at least contain, the systemic inflammation.

Although p.F8S is a protein-altering variant and is associated with immunological correlates, this study does not provide direct experimental evidence for altered IFNAR2 receptor function, ligand binding, signaling efficiency, or protein stability. Therefore, causality cannot be established, and functional observations should be interpreted as correlative.

In conclusion, the *IFNAR2* p.F8S missense variant is associated with predisposition to severe COVID-19 and an altered response to SARS-CoV-2 infection at the transcriptional level in immune cell populations. The impact of *IFNAR2* p.F8S on the differential composition of IFNAR2^+^ cell populations and the differential expression of anti- and pro-inflammatory cytokines support the hypothesis that the variant differentially modulates the IFNAR2-mediated response in DCs.

Overall, this study contributes to enhancing the general understanding of cytokine-related gene variants in infectious diseases and to assessing their role in monitoring the specific effects of pandemics across diverse populations.

## 4. Materials and Methods

### 4.1. Study Design and FOGS COHORT

All FOGS participants were recruited from a single geographic area and were of self-reported European ancestry; therefore, large-scale population stratification is unlikely, although subtle ancestry effects cannot be completely excluded.

Patients. FOGS cross sectional cohort includes 487 patients with severe COVID-19 defined as hospitalization with respiratory failure and a confirmed SARS-CoV-2 viral RNA RT-qPCR test from nasopharyngeal swabs or other relevant biologic fluids, from intensive care units and general wards [10] at the Fondazione IRCCS Cá Granda Ospedale Maggiore Policlinico, Milan. Data about disease severity were available for all the patients: among them, 188 required no intensive respiratory support, 202 presented mild respiratory damage, while 97 presented a severe phenotype, defined as the necessity to undergo invasive mechanical ventilation or in-hospital mortality. The main genetic and clinical description of the FOGS cohort was provided in Tables S10 and S11.

Healthy controls. We included as healthy controls 869 blood donors, retrieved from an independent case–control cohort of COVID-19 patients both locally and temporally matched to the cases during the first wave of infection at Fondazione IRCCS Ca’ Granda Ospedale Maggiore Policlinico of Milan; they were randomly selected and underwent genotyping for the purpose of the genetic association analysis of a previous study [6]. The main genetic description of the FOGS cohort was provided in Table S10.

### 4.2. Sample Processing, Genotyping, and Imputation

The final case–control data sets comprised 487 patients and 869 blood donors as controls. DNA extraction was performed using a Chemagic 360 (PerkinElmer; Waltham, MA, USA) with the use of the low-volume kit CMG-1491 and the buffy-coat kit CMG-714 (Chemagen Technologie GmbH, Baesweiler, Germany), respectively. For genotyping, the Global Screening Array (GSA), version 2.0 (Illumina, Inc., San Diego, CA, USA), which contains 712,189 variants, was used before quality control. Details on genotyping and quality-control procedures are described previously [10]. To maximize genetic coverage, we performed single-nucleotide polymorphism (SNP) imputation on genome build GRCh38 using the Michigan Imputation Server and 194,512 haplotypes generated by the Trans-Omics for Precision Medicine (TOPMed) program (freeze 5). A total of 8,965,091 SNPs were included in the cohort. The rs2229207 p.F8S *IFNAR2* variant was genotyped with GSA (MAF = 0.10111; R^2^ = 0.99819; ER^2^ = 0.92234; TYPED)

### 4.3. Validation in the FOGS Cohort

The main goal of the first part of the study was to increase the study power by reducing the number of variants tested (and therefore the denominator when correcting for multiplicity of testing), by selecting variants with a higher likelihood of being pathogenic. To accomplish this purpose, we focused on low-frequency missense/splice variants robustly associated with damage protein function, excluding from the dataset variants not reported all clinically associated with pathogenic phenotypes on the ClinVar database (version 20200316). The prioritized set of variants was employed for a genotype to phenotype correlations by generalized linear models using multivariate binomial logistic, ordinal logistic or linear modeling when appropriate. Although p.F8S is a low-frequency variant, association analyses were performed using logistic regression under an additive coding, a commonly adopted approach in GWAS, with results interpreted conservatively and supported by carrier-based analyses. Multiple-testing correction was applied only to the final set of 33 coding variants formally tested for association with severe COVID-19; upstream variant prioritization did not involve hypothesis testing and followed a previously published and validated framework [18]. Statistical analysis was performed using R version 4.0.3. Linkage disequilibrium analysis was carried out employing plink software, version 1.9. Replication analyses were performed using COVID-19 Host Genetics Initiative summary statistics, for which the population structure was addressed by the contributing studies using standard GWAS methodologies; individual-level data were not available for additional ancestry-adjusted analyses. For the replication of the association in a larger global cohort, the COVID-19 Host Genetics Initiative (HGI; release 7) was interrogated, considering the European group of patients employed in the meta-analysis. Conditional analysis was performed with SOJO R package (version 2.0) against the B2 HGI phenotype.

### 4.4. DNA Isolation and Whole Exome Sequencing (WES) of Two Patients with COVID-19 Who Underwent Lung-Transplantation

Genomic DNA (gDNA) was extracted from PBMCs of patients using QIAsymphony DSP DNA Midi kit (QIAGEN GmbH, Hilden, Germany) and quantified by Qubit instrumentation (Thermo Fisher Scientific Inc., Waltham, MA, USA). DNA libraries were enriched for exome sequencing by the SureSelect Clinical Research Exome v2 kit (Agilent, Santa Clara, CA, USA) and sequencing was subsequently performed on NextSeq2000 (Illumina, Inc., San Diego, CA, USA) at the Omics Science Laboratory, the genomic platform of our hospital Raw reads quality control was performed using FastQC software version 0.12.1 (Brabaham bioinformatics, Cambridge, UK). Reads were mapped on human GRCh38 genome using MEM algorithm of Burrows Wheeler Aligner (BWA) version 0.7.10; low quality alignments and duplicate reads were filtered out using SAMtools to generate high quality bam files. Mapping quality was checked using Picard-tools (http://broadinstitute.github.io/picard, accessed on 1 November 2025) and Bedtools. Variant calling was performed following Genome Analysis Toolkit (GATK) best; variant quality score log-odds (VQSLOD) above 99% tranche were considered true positives and variants present in <20% of total reads discarded. Indel left normalization was performed using BCFtools software version 1.21. For variant annotation both variant effect predictor (VEP) and ANNOVAR tools version 2023 were used, while VCFtools allowed the exclusion of variants over the VQSLOD threshold and variants called in less than 95% of samples.

The two COVID-19 patients who underwent bilateral lung transplantation due to irreversible respiratory failure presented a mean coverage of 86× (coverage 10×: 69%) and 72× (coverage 10×: 67%), respectively. As previously reported by Croci and Vaira et al. [13], patient 1 was an 18-year-old male while patient 2 was a 48-year-old male. Both patients were carriers for the rs657152 variant located in locus 9q34.2 responsible for at-risk ABO blood group (i.e., non-O group), with A+ and B+ group, respectively. Patients also resulted in non-carriers for the rs11385942 (A) variant top hit risk variant located in 3p21.31 locus and previously associated with pathology severity in the first COVID-19 genome-wide association study [10].

The immunopathological features of these patients have been already characterized in detail with a particular focus on their clinical history, SARS-CoV-2 viral load, histopathology and the expression profile of a selected set of genes in lungs and PBMC samples [13].

### 4.5. Clinical and Virological Data

Demographic and clinical data were collected, including sex, age, BMI, comorbidities, maintenance therapy, presence of acute symptoms and their duration, need of hospitalization, admission to intensive care unit (ICU), need of oxygen supplementation and non-invasive or invasive ventilation. Nasopharyngeal swab specimens were collected by the local virology unit at Fondazione IRCCS Ca’ Granda Ospedale Maggiore Policlinico, Milan, Italy to detect SARS-CoV-2. The presence of SARS-CoV-2 in respiratory specimens was detected by real-time reverse transcription (RT-PCR) methods. Primers and probes targeting the envelope gene were used. Conditions for the amplifications were 50 °C for 15 min, 95 °C for 3 min, followed by 45 cycles of 95 °C for 15 s and 60 °C for 30 s. Input of clinical and virological data was accomplished using the REDCap electronic data capture tool, hosted by Fondazione IRCCS Ca’ Granda Ospedale Maggiore Policlinico. For the association analysis for the need of intensive respiratory support during hospitalization, we considered high flux oxygen supplementation, mechanical ventilation, extracorporeal continuous membrane oxygenation to define different degrees of severity (none, *n* = 188; mild, *n* = 202; sustained, *n* = 97). Severe COVID-19 was defined as the need for hospitalization for respiratory failure or other medical complications, whereas intensive respiratory support (need of invasive ventilation, or continuous positive airway pressure ventilation, or high oxygen flux ventilation) was evaluated as a secondary outcome reflecting disease progression after admission.

### 4.6. PBMC Isolation from SARS-CoV-2-Infected Patients

Peripheral blood was collected in EDTA vacutainers at the time of the hospitalization, then PBMCs were isolated following the standard protocols. Briefly, vacutainers were centrifuged for 10 min at 20 °C at 1560× *g* and buffy coat collected to be diluted up to 10 mL with 0.9% saline. Then, the suspension was layered on 3 mL of Ficoll to let the cell percolate at 900× *g* for 20 min at 20 °C in brake off setting. PBMCs were finally collected in 10 mL Falcon tube, and washed twice in 10 mL of saline through centrifugation at 450× *g* and 350× *g*, respectively. Isolated cells were frozen in 1 mL of 10% DMSO FBS cold freezing medium.

### 4.7. Transcriptomic Analysis

Bulk RNA-Sequencing. Fifty-seven patients with severe disease were randomly selected from the FOGS cohort (*n* = 9 IFNAR2 p.F8S carriers) and their PBMCs, biobanked at the Fondazione IRCCS Cá Granda Ospedale Maggiore Policlinico, were employed for bulk RNA-Seq experiment. Trizol reagent (Life Technologies) was employed to extract RNA according to the manufacturer’s protocol. Once libraries were enriched with TruSeq RNA Exome, which provides high capture efficiency focusing sequencing efforts on the higher content of RNA coding regions, RNA was sequenced in paired-end mode employing P3 cartridges and NextSeq2000 (both from Illumina). Reads were mapped by a custom pipeline, encompassing reads’ quality check (FastQC software, Babraham Bioinformatics, Cambridge, UK), on GRCh38 reference genome (ENSEMBL human transcript reference assembly v99) by STAR mapper (version 2.7.11b). Reads count was performed using RSEM package (version 1.3.3). Counts were normalized using DESeq2 package (version 1.44.0). Batch effects in RNA-seq data were assessed and corrected using standard normalization and batch-correction procedures prior to differential expression analysis provided by RUVSeq R package (version 1.38.0), applying the RUVg function.

For principal component analysis (PCA), gene-level expression data were normalized under the null model through the DESeq2 standard pipeline, and variance stabilizing transformation function was applied. Differential expression analyses were performed using DESeq2, with models adjusted for biological sex, age, carriage of the rs11385942 (LZTFL1) risk variant, ABO blood group O status, and lymphocyte-to-monocyte ratio (LMR) to account for potential confounding related to immune cell composition. To identify differentially expressed pathways, Ingenuity Pathway Analysis (IPA, winter 2024 release, Qiagen: www.qiagen.com/ingenuity) software was employed on differentially expressed or significantly correlated genes. To identify enriched gene ontology (GO) terms in overrepresentation analysis, we queried the Panther database considering significant (FDR < 0.05) differentially expressed genes presenting a log_2_ fold-change greater than 1 or less than −1 in carriers vs. non-carriers of IFNAR2 p.F8S.

### 4.8. scRNA-Sequencing

Eight independent patients from two different scRNA-Seq experiments have been selected to validate the role of p.F8S in DCs during COVID-19; samples nCoV3, nCoV5, nCoV7, nCoV8, nCoV9 and nCoV10 have been selected from Lee, J.S. et al. [22], while samples COVID_555 and COVID_558 have been selected from Wilk, A. J. et al. [23], under accession numbers GSE149689 and GSE150728, respectively. Fastq files of all the samples composing the respective experiments have been aligned with STAR and then the .bam file used to call p.F8S IFNAR2 genotype using Monopogen [24] software. For samples where the variant could not be called, the p.F8S genotype was imputed using the TOPMed Imputation Server and the sample considered whereas the variant had a non-null R^2^ value. For data analysis, Seurat v5 was employed in R environment v4.4.1. Briefly, sample data were pre-processed in order to filter out cells with a number of transcripts lower than 200 or higher than 5000, percentage of mitochondrial and ribosomal genes higher than 10% and then samples were merged in a unique Seurat object for data normalization, scaling and dimensionality reduction (PCA) prior to integration. After filtering, we retained 15,552 cells for wild-type patients (*n* = 2), 10,433 for heterozygous patients (*n* = 3) and 1101 for homozygous patients (*n* = 3). Canonical correlation analysis has been used to integrate the layers of the Seurat object and a cut-off of 0.2 was used to find cell clusters. Positive markers for each cluster have been used for manually curated cell type annotation with the aid of EnrichR software (version version 3.4) (https://maayanlab.cloud/Enrichr, accessed on 1 November 2025). For differential expression analysis for each immune population subset, DESeq2 was employed and significant genes (FDR < 0.05) used to run GSEA (http://www.broad.mit.edu/gsea, accessed on 1 November 2025).

### 4.9. IFN Treatment and RT-qPCR

All experiments shown in Figure S3 and Table S7 were performed on primary cells isolated from individuals enrolled in the FOGS cohort. No plasmid transfection, viral transduction, or overexpression of IFNAR2 variants was performed. PBMCs were seeded (1 × 10^5^ per well) in 6-well attachment plates (Corning Inc., Corning, NY, USA) and cultured for 48 h in RPMI medium supplemented with L-glutamine (Sigma-Aldrich, St. Louis, MO, USA), 10% FBS (Thermo Fisher Scientific, Waltham, MA, USA) and 1% penicillin/streptomycin (Sigma-Aldrich, St. Louis, MO, USA) at 37 °C, 5% CO_2_. Then, culture medium was replaced, taking care of the cells in suspension that were collected and re-plated with fresh culture medium on the attached cells.

Cells were then treated with IFN-α (Thermo Fisher Scientific, Waltham, MA, USA) at a concentration of 10 µg/mL in order to mimic the cytokine storm characterizing COVID-19 and were harvested at 1 h and 4 h to investigate the cell response at transcriptional level. RNA was extracted as described above. Superscript VILO cDNA synthesis kit (Life Technologies, Carlsbad, CA, USA) was used for the retro-transcription of total RNA, then, qPCR was used to evaluate gene expression exploiting SYBR Green chemistry (Fast SYBR Green Master Mix; Life Technologies). Cells were analyzed according to endogenous *IFNAR2* genotype, and comparisons reflect differences in individual-derived samples. The relative gene expression of the genes implicated in the IFNAR2 signal transduction (*IFNAR2*, the receptor; *STAT2*, the main signal transducer; *ISG15* the main effector) was measured by RT-qPCR and analyzed via generalized linear regression (*p*-values reflect the regression coefficient associated with p.F8S genotype). The list of primers used for the experiment is provided in Table S12.

### 4.10. IFNAR2 Cell Surface Evaluation on Healthy PBMCs

Among the healthy blood donors of the Fondazione IRCCS Ca’ Granda Ospedale Maggiore Policlinico, eight participants were selected and considered in this experiment. IFNAR2 rs2229207 genotype was assessed via TaqMan assay (ThermoFisher); three participants were heterozygous for the variants, two homozygous and three did not present the variant.

Peripheral blood was stained within 2 h from collection. IFNAR2 expression was evaluated on circulating cells using two flow cytometry panels, consisting of two cocktails of the following anti-human fluorochrome-conjugated monoclonal antibodies: FITC-CD3, PE-IFNAR2, PerCP-Cy5.5-CD16, PE-Cy7-CD14, APC-CD4, APC-Cy7-CD8, APC-R700-CD19, V450-CD56, and BV605-CD45 (for lymphocytes, monocytes and neutrophils), and FITC-Lineage-1 (including a mix of anti-CD3, -CD14, -CD16, -CD19, -CD20, and -CD56), PE-IFNAR2, PerCP-Cy5.5 HLA-DR, APC-CD11c, BV510-CD123, and BV605-CD45 (for dendritic cells). Erythrocytes were removed with a lyse-and-wash protocol with ammonium chloride, then samples were acquired with the FACSLyric flow cytometer (BD Biosciences, Franklin Lakes, NJ, USA) and analyzed with FlowJo version 10.10. Blood count was used to obtain absolute count of cell populations. Monoclonal antibodies were properly titrated, and cytometer and compensation matrix were daily calibrated with CS&T beads. Fluorescence Minus One (FMO) for the PE channel was performed to set IFNAR2 gate for downstream calculation of the mean fluorescence intensity variation (ΔMFI) for each population subset. All reagents, instrument and software were from BD (Becton Dickinson Biosciences, BD, Franklin Lakes, NJ, USA), except the IFNAR2 antibody from Miltenyi Biotec (Miltenyi Biotec, Bergisch Gladbach, Germany).

### 4.11. Statistics

Continuous variables were normalized through rank-based inverse normal transformation (RINT) and used to test associations in multivariable linear models. For dichotomous variables, multivariable logistic regression was applied. Data analysis and statistical comparisons were performed with R version 4.4.1 (cran.r-project.org). Data visualization was performed either with GraphPad Prism 8.0 or with R.

## Figures and Tables

**Table 1:** *IFNAR2* p.F8S variant association with COVID-19 is independent of top hit variants highlighted in previous GWAS.

	Model 1		Model 2		Model 3		Model 4	
Variable	*p*	OR (95% C.I)	*p*	OR (95% C.I)	*p*	OR (95% C.I)	*p*	OR (95% C.I)
p.F8S rs2229207	0.032	1.31 (1.02–1.68)	0.001	1.76 (1.26–2.45)	0.001	1.73 (1.24–2.42)	0.017	1.60 (1.09–2.34)
Age	-	-	<0.001	1.31 (1.11–1.14)	<0.001	1.13 (1.11–1.15)	<0.001	1.13 (1.11–1.14)
Sex, Male	-	-	0.688	1.07 (0.78–1.47)	0.727	1.06 (0.77–1.46)	0.700	1.07 (0.77–1.47)
rs11385942	-	-	-	-	<0.001	1.89 (1.37–2.63)	<0.001	1.88 (1.36–2.61)
O group	-	-	-	-	0.053	0.74 (0.54–1.00)	0.054	0.74 (0.54–1.01)
rs2236757	-	-	-	-	-	-	0.668	1.13 (0.65–1.94)
rs13050728	-	-	-	-	-	-	0.644	0.74 (0.20–2.70)
rs9636867	-	-	-	-	-	-	0.912	0.93 (0.27–3.21)

## Data Availability

All data relevant to the study are included in the article or uploaded as supplementary information. The data that support the findings of this study are available on request from the corresponding author.

## Supplementary Materials

The following supporting information can be downloaded at: https://www.mdpi.com/article/10.3390/ijms27020992/s1. References [27,28,29,30,31,32,33,34,35,36] was cited in Supplementary Materials.

## Abbreviations

The following abbreviations are used in this manuscript:

SARS-CoV-2	Severe acute respiratory syndrome coronavirus 2
COVID-19	Coronavirus disease 2019
FOGS	Fondazione Genomic Study
C.I.	Confidence Interval
IFNAR2	Interferon α/β receptor subunit 2
IFNAR1	Interferon α/β receptor subunit 1
WES	Whole exome sequencing
IL10RB	Interleukin-10 receptor subunit β
GWAS	Genome-wide association study
PBMCs	Peripheral Blood Mononuclear Cells
MAF	Minor allele frequency
NFE	Non-Finnish European
SNPs	Single nucleotide polymorphisms
NLR	Neutrophil to lymphocyte ratio
CRP	C-reactive protein
IL-6	Interleukin-6
LZFTL1	Leucine zipper transcription factor like 1
LMR	Lymphocyte-to-Monocyte Ratio
FUT2	Fucosyltransferase 2
PCA	Principal component analysis
CD81	Cluster of Differentiation 81
DEG	Differentially expressed gene
TERMs	Tetraspanin-enriched microdomains
CD19	Cluster of Differentiation 81
PD-1	Programmed cell death protein 1
PD-L1	Programmed cell death protein ligand 1
ORA	Overrepresentation analysis
MHC	Major histocompatibility complex
IFN	Interferon
C4B	Complement C4B
STAT2	Signal transducer and activator of transcription 2
ISG15	Interferon-stimulated gene 15
CD4	Cluster of differentiation 4
CD8	Cluster of differentiation 8
mDC	Myeloid dendritic cell
pDC	Plasmacytoid dendritic cells
GSEA	Gene set enrichment analysis

## References

[1] Zhu N., Zhang D., Wang W., Li X., Yang B., Song J., Zhao X., Huang B., Shi W., Lu R. (2020). A Novel Coronavirus from Patients with Pneumonia in China, 2019. N. Engl. J. Med..

[2] Lavezzo E., Franchin E., Ciavarella C., Cuomo-Dannenburg G., Barzon L., Del Vecchio C., Rossi L., Manganelli R., Loregian A., Navarin N. (2020). Suppression of a SARS-CoV-2 outbreak in the Italian municipality of Vo’. Nature.

[3] Huang C., Wang Y., Li X., Ren L., Zhao J., Hu Y., Zhang L., Fan G., Xu J., Gu X. (2020). Clinical features of patients infected with 2019 novel coronavirus in Wuhan, China. Lancet.

[4] Chen G., Wu D., Guo W., Cao Y., Huang D., Wang H., Wang T., Zhang X., Chen H., Yu H. (2020). Clinical and immunological features of severe and moderate coronavirus disease 2019. J. Clin. Investig..

[5] Varga Z., Flammer A.J., Steiger P., Haberecker M., Andermatt R., Zinkernagel A.S., Mehra M.R., Schuepbach R.A., Ruschitzka F., Moch H. (2020). Endothelial cell infection and endotheliitis in COVID-19. Lancet.

[6] Feldstein L.R., Rose E.B., Horwitz S.M., Collins J.P., Newhams M.M., Son M.B.F., Newburger J.W., Kleinman L.C., Heidemann S.M., Martin A.A. (2020). Multisystem Inflammatory Syndrome in U.S. Children and Adolescents. N. Engl. J. Med..

[7] Gorog D.A., Storey R.F., Gurbel P.A., Tantry U.S., Berger J.S., Chan M.Y., Duerschmied D., Smyth S.S., Parker W.A.E., Ajjan R.A. (2022). Current and novel biomarkers of thrombotic risk in COVID-19: A Consensus Statement from the International COVID-19 Thrombosis Biomarkers Colloquium. Nat. Rev. Cardiol..

[8] Pairo-Castineira E., Clohisey S., Klaric L., Bretherick A.D., Rawlik K., Pasko D., Walker S., Parkinson N., Fourman M.H., Russell C.D. (2021). Genetic mechanisms of critical illness in COVID-19. Nature.

[9] Valenti L., Griffini S., Lamorte G., Grovetti E., Renteria S.C.U., Malvestiti F., Scudeller L., Bandera A., Peyvandi F., Prati D. (2021). Chromosome 3 cluster rs11385942 variant links complement activation with severe COVID-19. J. Autoimmun..

[10] Ellinghaus D., Degenhardt F., Bujanda L., Buti M., Albillos A., Invernizzi P., Prati D., Baselli G., Asselta R., Severe COVID-19 GWAS Group (2020). Genomewide Association Study of Severe COVID-19 with Respiratory Failure. N. Engl. J. Med..

[11] (2020). COVID-19 Host Genetics Initiative. The COVID-19 Host Genetics Initiative, a global initiative to elucidate the role of host genetic factors in susceptibility and severity of the SARS-CoV-2 virus pandemic. Eur. J. Hum. Genet. EJHG.

[12] (2023). COVID-19 Host Genetics Initiative. A second update on mapping the human genetic architecture of COVID-19. Nature.

[13] Croci G.A., Vaira V., Trabattoni D., Biasin M., Valenti L., Baselli G., Barberis M., Guerini Rocco E., Gregato G., Scandroglio M. (2021). Emergency Lung Transplantation after COVID-19: Immunopathological Insights on Two Affected Patients. Cells.

[14] (2021). COVID-19 Host Genetics Initiative. Mapping the human genetic architecture of COVID-19. Nature.

[15] Smieszek S.P., Polymeropoulos V.M., Xiao C., Polymeropoulos C.M., Polymeropoulos M.H. (2021). Loss-of-function mutations in IFNAR2 in COVID-19 severe infection susceptibility. J. Glob. Antimicrob. Resist..

[16] Liu B.M., Hill H.R. (2020). Role of Host Immune and Inflammatory Responses in COVID-19 Cases with Underlying Primary Immunodeficiency: A Review. J. Interferon Cytokine Res. Off. J. Int. Soc. Interferon Cytokine Res..

[17] Pelusi S., Baselli G., Pietrelli A., Dongiovanni P., Donati B., McCain M.V., Meroni M., Fracanzani A.L., Romagnoli R., Petta S. (2019). Rare Pathogenic Variants Predispose to Hepatocellular Carcinoma in Nonalcoholic Fatty Liver Disease. Sci. Rep..

[18] Baselli G.A., Jamialahmadi O., Pelusi S., Ciociola E., Malvestiti F., Saracino M., Santoro L., Cherubini A., Dongiovanni P., Maggioni M. (2022). Rare ATG7 genetic variants predispose patients to severe fatty liver disease. J. Hepatol..

[19] Degenhardt F., Ellinghaus D., Juzenas S., Lerga-Jaso J., Wendorff M., Maya-Miles D., Uellendahl-Werth F., ElAbd H., Rühlemann M.C., Arora J. (2022). Detailed stratified GWAS analysis for severe COVID-19 in four European populations. Hum. Mol. Genet..

[20] New C., Lee Z.Y., Tan K.S., Wong A.H.P., Wang D.Y., Tran T. (2021). Tetraspanins: Host Factors in Viral Infections. Int. J. Mol. Sci..

[21] Colonna M., Trinchieri G., Liu Y.J. (2004). Plasmacytoid dendritic cells in immunity. Nat. Immunol..

[22] Lee J.S., Park S., Jeong H.W., Ahn J.Y., Choi S.J., Lee H., Choi B., Nam S.K., Sa M., Kwon J.-S. (2020). Immunophenotyping of COVID-19 and influenza highlights the role of type I interferons in development of severe COVID-19. Sci. Immunol..

[23] Wilk A.J., Rustagi A., Zhao N.Q., Roque J., Martínez-Colón G.J., McKechnie J.L., Ivison G.T., Ranganath T., Vergara R., Hollis T. (2020). A single-cell atlas of the peripheral immune response in patients with severe COVID-19. Nat. Med..

[24] Dou J., Tan Y., Kock K.H., Wang J., Cheng X., Tan L.M., Han K.Y., Hon C.-C., Park W.-Y., Shin J.W. (2024). Single-nucleotide variant calling in single-cell sequencing data with Monopogen. Nat. Biotechnol..

[25] Quast T., Eppler F., Semmling V., Schild C., Homsi Y., Levy S., Lang T., Kurts C., Kolanus W. (2011). CD81 is essential for the formation of membrane protrusions and regulates Rac1-activation in adhesion-dependent immune cell migration. Blood.

[26] Ali S., Mann-Nüttel R., Schulze A., Richter L., Alferink J., Scheu S. (2019). Sources of Type I Interferons in Infectious Immunity: Plasmacytoid Dendritic Cells Not Always in the Driver’s Seat. Front. Immunol..

[27] Park Y.J., Acosta D., Vassell R., Tang J., Khurana S., Weiss C.D., Golding H., Zaitseva M. (2022). D-dimer and CoV-2 spike-immune complexes contribute to the production of PGE2 and proinflammatory cytokines in monocytes. PLoS Pathog..

[28] Zhang H., Wu H., Pan D., Shen W. (2022). D-dimer levels and characteristics of lymphocyte subsets, cytokine profiles in peripheral blood of patients with severe COVID-19: A systematic review and meta-analysis. Front. Med..

[29] Valenti L., Bergna A., Pelusi S., Facciotti F., Lai A., Tarkowski M., Berzuini A., Caprioli F., Santoro L., Baselli G. (2021). SARS-CoV-2 seroprevalence trends in healthy blood donors during the COVID-19 outbreak in Milan. Blood Transfus..

[30] Bruchez A., Sha K., Johnson J., Chen L., Stefani C., McConnell H., Gaucherand L., Prins R., Matreyek K.A., Hume A.J. (2020). MHC class II transactivator CIITA induces cell resistance to Ebola virus and SARS-like coronaviruses. Science.

[31] Cui J., Zhu L., Xia X., Wang H.Y., Legras X., Hong J., Ji J., Shen P., Zheng S., Chen Z.J. (2010). NLRC5 Negatively Regulates the NF-κB and Type I Interferon Signaling Pathways. Cell.

[32] Chang K.T., Wu H.J., Liu C.W., Li C.Y., Lin H.Y. (2022). A Novel Role of Arrhythmia-Related Gene KCNQ1 Revealed by Multi-Omic Analysis: Theragnostic Value and Potential Mechanisms in Lung Adenocarcinoma. Int. J. Mol. Sci..

[33] Rui X., Shao S., Wang L., Leng J. (2019). Identification of recurrence marker associated with immune infiltration in prostate cancer with radical resection and build prognostic nomogram. BMC Cancer.

[34] Gao T., Zhu L., Liu H., Zhang X., Wang T., Fu Y., Li H., Dong Q., Hu Y., Zhang Z. (2022). Highly pathogenic coronavirus N protein aggravates inflammation by MASP-2-mediated lectin complement pathway overactivation. Signal Transduct. Target. Ther..

[35] Dai Y., Wang J., Jeong H.H., Chen W., Jia P., Zhao Z. (2021). Association of CXCR6 with COVID-19 severity: Delineating the host genetic factors in transcriptomic regulation. Hum. Genet..

[36] Mahesh G., Biswas R. (2019). MicroRNA-155: A Master Regulator of Inflammation. J. Interferon Cytokine Res..

