# Supplementary material for: IFNAR2 p.F8S Variant Associates with Severe COVID-19 and Adaptive Immune Cell Activation Modulation
Source: Int J Mol Sci. 2026 Jan 19;27(2):992. doi: 10.3390/ijms27020992 (PMC12841681; doi:10.3390/ijms27020992)
Supplement: Supplementary file 1 [file ijms-27-00992-s001.zip › Malvestiti et al. [Supplementary].pdf]

International Journal of *Molecular Sciences*

MDPI

Article

IFNAR2 p.F8S Variant Associates with Severe COVID-19 and Adaptive Immune Cell Activation Modulation

Francesco Malvestiti [1,2], Angela Lombardi [2], Francesco Gentile [3], Veronica Torcianti [2], Elena Trombetta [4], Alessandro Cherubini [2], Giuseppe Lamorte [2], Sara Colonia Uceda Renteria [5], Daniele Marchelli [1], Lorenzo Rosso [1,6], Alessandra Bandera [7], Flora Peyvandi [1,8], Francesco Blasi [1,9], Giacomo Grasselli [1,10], Laura Porretti [4], Saleh Alqahtani [11,12], Daniele Prati [2], Roberta Gualtierotti [1,8], Blagoje Soskic [13], Valentina Vaira [1,3], Luisa Ronzoni [2] and Luca Valenti [1,2,*]

1 Department of Pathophysiology and Transplantation, Università degli Studi di Milano, 20122 Milan, Italy; francesco.malvestiti@unimi.it (F.M.); daniele.marchelli@unimi.it (D.M.); lorenzo.rosso@policlinico.mi.it (L.R.); flora.peyvandi@unimi.it (F.P.); francesco.blasi@policlinico.mi.it (F.B.); giacomo.grasselli@policlinico.mi.it (G.G.); roberta.gualtierotti@unimi.it (R.G.); valentina.vaira@unimi.it (V.V.)
2 Precision Medicine Lab, Transfusion Medicine and Hematology, Biological Resource Centre, Fondazione IRCCS Ca' Granda Ospedale Maggiore Policlinico, 20122 Milan, Italy; angela.lombardi@policlinico.mi.it (A.L.); veronica.torcianti@policlinico.mi.it (V.T.); alessandro.cherubini@policlinico.mi.it (A.C.); giuseppe.lamorte@policlinico.mi.it (G.L.); daniele.prati@policlinico.mi.it (D.P.); luisa.ronzoni@policlinico.mi.it (L.R.)
3 Division of Pathology, Fondazione IRCCS Ca' Granda Ospedale Maggiore Policlinico, 20122 Milan, Italy; francesco.gentile@meduniwien.ac.at
4 Flow Cytometry Laboratory, Clinical Pathology, Fondazione IRCCS Ca' Granda Ospedale Maggiore Policlinico; 20122 Milan, Italy; elena.trombetta@policlinico.mi.it (E.T.); laura.porretti@policlinico.mi.it (L.P.)
5 Virology Unit, Fondazione IRCCS Ca' Granda Ospedale Maggiore Policlinico, 20122 Milan, Italy; sara.ucedarenteria@policlinico.mi.it
6 Thoracic Surgery and Lung Transplantation Unit, Fondazione IRCCS Cà Granda Ospedale Maggiore Policlinico, 20122 Milan, Italy
7 Infectious Diseases Unit, Fondazione IRCCS Ca' Granda Ospedale Maggiore Policlinico, 20122 Milan, Italy; alessandra.bandera@policlinico.mi.it
8 SC Medicine-Haemostasis and Thrombosis, Fondazione IRCCS Ca' Granda Ospedale Maggiore Policlinico, 20122 Milan, Italy
9 Respiratory Unit and Cystic Fibrosis Center, Fondazione IRCCS Cà Granda Ospedale Maggiore Policlinico 20122 Milan, Italy
10 Department of Anesthesia, Critical Care and Emergency, Foundation IRCCS Ca' Granda Maggiore Policlinico Hospital, 20122 Milan, Italy
11 Liver, Digestive, and Lifestyle Health Research Section, and Organ Transplant Center of Excellence, King Faisal Specialist Hospital & Research Center, Riyadh 11211, Saudi Arabia; salqaht1@jhmi.edu
12 Division of Gastroenterology and Hepatology, Weill Cornell Medicine, New York, NY 10065, USA
13 Human Technopole, Viale Rita Levi-Montalcini 1, 20157, Milan, Italy; blagoje.soskic@fht.org
* Correspondence: luca.valenti@unimi.it

1. Supplementary Results

S2.1 Impact of IFNAR2 rs2229207 T>C p.F8S on clinical and virological features

To assess if the IFNAR2 p.F8S variant may play a role in coagulopathy due to coagulation activation contributing to aberrant cytokine responses following SARS-CoV2 infection [1,2], we tested the association between the variant and circulating levels of D-Dimer. By multivariable linear regression, adjusted for age, biological treatment and steroid treatment, we found p.F8S variant did not associate with increased levels of D-dimer in COVID-19 patients (Table S4, carriers=55 vs non carriers=233, p=0.986), indicating that the enhanced inflammation following SARS-Cov-2 infection was decoupled from thrombotic events.

At a sensitivity analysis to evaluate the relationship between carriage of the variant and patients' comorbidities, we found that the presence of a known immunodeficiency (n=42) was inversely associated with IFNAR2 p.F8S variant (Table S13, p=0.047, OR=0.30, 95% C.I.=0.09-0.98), suggesting that the predisposition to severe COVID-19 was limited to those individuals able to support immune response upon infection. In addition, to explore possible mechanisms mediated by the variant in modulating immune response, we evaluated its relationship with circulating levels of SARS-CoV-2 antibodies. We analyzed by multivariable regression the association between the carriage of p.F8S variant and anti-SARS-CoV-2 IgG titer in the Covid-19 Donors Study (CODS) cohort [3], adjusted for age, gender, BMI and O blood group. No difference in circulating titer was observed in carriers (n=52) vs non carriers (n=205) (Table S4, p=0.60) even when only IgM-positive patients were considered in the analysis (Table S4, carriers=13 vs non-carriers=50, p=0.49). These results do not support a direct impact of the p.F8S variant on the production of higher titers of SARS-CoV-2 antibodies.

To evaluate whether IFNAR2 p.F8S could influence COVID-19 severity and inflammation by influencing SARS-CoV-2 replication, we analyzed by multivariable regression the impact on SARS-CoV-2 viral load as detected by nucleocapsid RNA levels in 242 patients without immunodeficiency, whose SARS-CoV-2 viral RNA polymerase-chain-reaction (RT-qPCR) data of nasopharyngeal swabs test at the time of the hospitalization were available (Table S14). No genotype-dependent differential expression of the nucleocapsid gene was detected in the univariable analysis (Figure S1).

S2.2 p.F8S associates with differential expression of immune genes in PBMCs transcriptomic profiles of patients with severe COVID-19

The main transcriptomic analysis highlighted the lack of *IFNAR2* differential expression between p.F8S carriers and non-carriers. Moreover, the whole *IFNAR2-STAT2-ISG15* axis composing the type I IFN signaling did not show any appreciable perturbation at the transcription level in the carriers of the p.F8S variant at the peak of SARS-CoV-2 infection (Supplementary Figure 2A).

In addition, the analysis revealed an upregulation in class I MHC genes, such as *HLA-B* (log$_2$-fold change=2.15, p=5.34*10-8, FDR=3.85*10-5), *HLA-F* (log$_2$-fold change=2.34, p=8.75*10^{-8}, FDR=5.21*10^{-5}), *HLA-E* (log$_2$-fold change=2.00, p=3.02*10^{-6}, FDR=4*10^{-4}), *HLA-C* (log$_2$-fold change=1.90, p=5.01*10^{-6}, FDR=5*10^{-4}), and *HLA-A* (log$_2$-fold change=1.81, p=1.21*10^{-6}, FDR=2*10^{-4}) as well as in class II MHC related genes *CIITA* (log$_2$-fold change=1.80, p=1.56*10^{-5}, FDR=1.15*10^{-3}) and *NLRC5* (log$_2$-fold change=1.27, p=9.32*10^{-4}, FDR=0.015) which are involved in antiviral mechanisms against coronaviruses, in the regulation of both cytokine response and type I interferon signaling pathways [4,5](Table S6). An appreciable upregulation was found in genes correlated with the activity of the immune profile of the host, such as *KCNQ1* (log$_2$-fold change=1.87, p=3.42*10^{-8}, FDR=3.38*10^{-5}) and *NDUFA13* (log$_2$-fold change=3.37, p=4.66*10^{-9}, FDR=9.87*10^{-6}) whose overexpression was previously associated with immunoactive profile of immune infiltration and immunomodulators [6,7], *FAM156A* (log$_2$-fold change=2.34, p=5.56*10^{-9}, FDR=1.03*10^{-5})(Figure 2B) and in *C4B* (log$_2$-fold change=3.03, p=3.43*10^{-7}, FDR=1.18*10^{-4}) genes (Figure 2F), which regulates complement activation whose dysregulation has been implicated in the development of acute lung diseases induced by highly pathogenic viruses [8].

Notably, among the most downregulated top significant DEGs, beyond a group of gene mainly involved in cell cycle, such as *CREG1*, *ENSA*, *SCML2*, *AVPI1* (Figure 2B), also *CXCR6*, (log$_2$-fold change=-2.50, p=2.41*10^{-7}, FDR=9.81*10^{-5}) a marker of tissue resident CD8$^+$ T cells whose downregulation was assessed in severe COVID-19 cases [9], *PCNA* (log$_2$-fold change=-1.71, p=6.75*10^{-5}, FDR=2.76*10^{-3}) and *CDKN2B* (log$_2$-fold change=-2.49,

https://doi.org/10.3390/ijms27020992

$p=3.40*10^{-5}$, $FDR=1.80*10^{-3}$) genes, known to be directly correlated to cell growth and cell cycle, were significantly downregulated in carriers of the p.F8S variant (Table S6).

Since IFNAR2 p.F8S was associated with changes in *IL10* signaling, we finally sought to determine potential changes in expression patterns of other interleukins by differential expression analysis. The p.F8S variant was negatively associated with the expression of *IL10* gene (p=0.007) (Figure 2F) and the correction for other signals at the *IFNAR2* locus did not attenuate this association (Figure S4A). Furthermore, *IL32* was the most overexpressed circulating-interleukin coding gene in carriers of the p.F8S variant at the peak of SARS-CoV-2 infection (p=0.043) (Figure 2F), suggesting that variations in the *IFNAR2* locus may contribute to the modulation of immune response via differential modulation of the expression pattern of anti- and pro-inflammatory cytokines, independently from other variants in the same gene (Figure S4A).

Consistent with these observations, IPA upstream regulator analysis also predicted the activation of cytokines, enzymes and transcription factors involved in different mechanisms of immune response and inflammation such as interferon-γ (*IFNG*), interleukin-27 (*IL-27*), DNA methyltransferase 3 alpha (*DNMT3A*), glutathione S-transferase omega 1 (*GSTO1*), cyclin dependent kinase inhibitor 2A (*CDKN2A*), tumor protein p53 (*TP53*) and lysine demethylase 5B (*KDM5B*) (Figure S4A). Furthermore, *mir-155*, a master regulator of inflammatory diseases, including cancer and pulmonary disorders [10], was predicted to be activated in the carriers of p.F8S variant (Figure S4B; activation z-score=1.98).

2. Supplementary Material

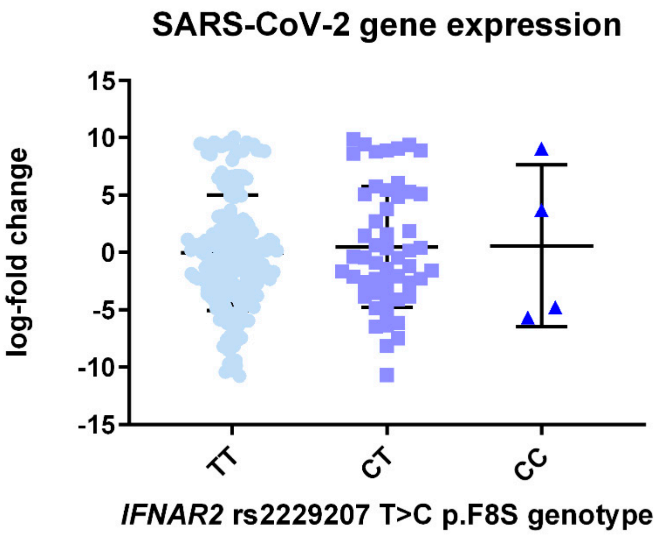

Figure S1. IFNAR2 p.F8S variant genotype does not associate to any enhancement of viral load in COVID-19 patients at the peak of infection.

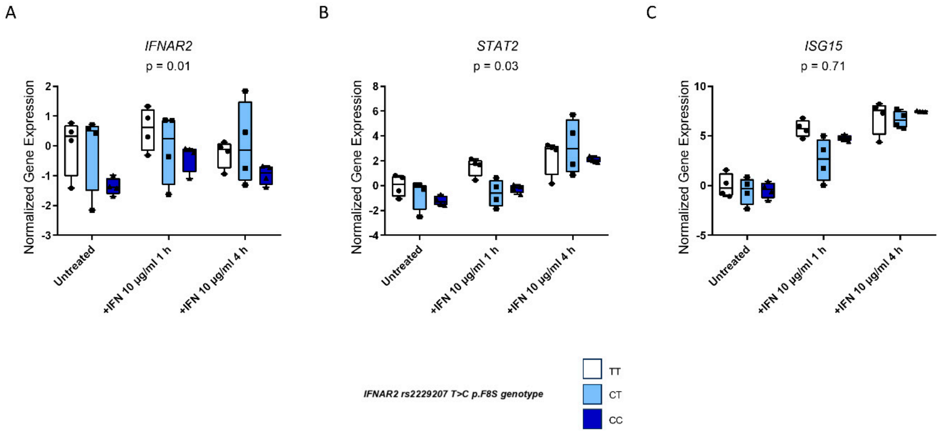

Figure S2. Comparison of IFNAR2, IFNAR1 and their ratio in COVID-19 patients at the peak of infection. IFNAR2 p.F8S variant carriage did not associate either to total IFNAR2 (**A**) or IFNAR1 (**B**) expression in total PBMCs, but it associated with IFNAR1/IFNAR2 ratio (**C**).

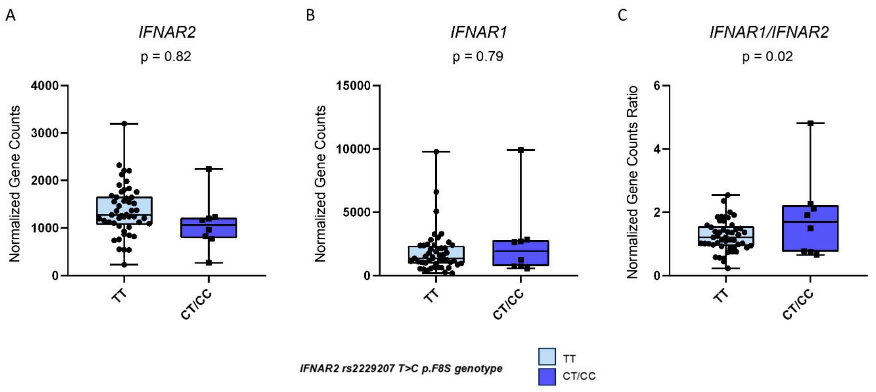

Figure S3. IFN-α treatment on healthy PBMCs. Relative gene expression levels of IFNAR2, STAT2, and ISG15 were measured by RT-qPCR in PBMCs from FOGS participants and analyzed using generalized linear regression models. Reported p-values correspond to the comparison between individuals with different p.F8S genotypes, for each gene. IFNAR2 mRNA levels did not show a significant transcriptional variation in response to IFN-α stimulation, but they are negatively associated with the IFNAR2 p.F8S variant carriage (**A**). STAT2 transcription showed the same association relative to the p.F8S variant genotype although it was upregulated in response to IFN-α stimulation (**B**) as ISG15, which however did not present any association with p.F8S variant (**C**).

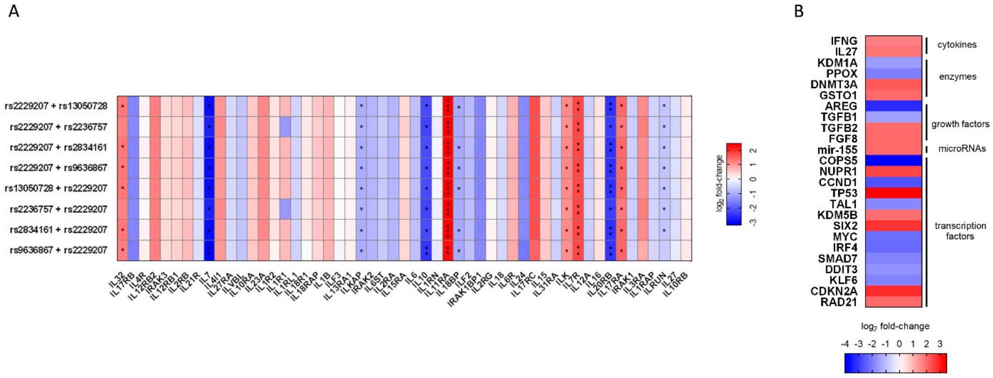

Figure S4. A. Schematic representation of model clustering: the correction for intronic IFNAR2 rs13050728 variant of the correlation between p.F8S variant and gene expression in PBMCs did not attenuate the association for IL-10 and IL-32 genes in COVID-19 patients carrying the p.F8S variant.

https://doi.org/10.3390/ijms27020992

B. IPA predicted upstream regulators. IPA upstream regulator analysis predicted the activation of cytokines, enzymes and transcription factors involved in different mechanisms of immune response and inflammation.

References

1. Park, Y. J. et al. D-dimer and CoV-2 spike-immune complexes contribute to the production of PGE2 and proinflammatory cytokines in monocytes. PLoS Pathog. 2022;18(4):e1010468.
2. Zhang, H. et al. D-dimer levels and characteristics of lymphocyte subsets, cytokine profiles in peripheral blood of patients with severe COVID-19: A systematic review and meta-analysis. Front Med. 2022;9:988666.
3. Valenti, L. et al. SARS-CoV-2 seroprevalence trends in healthy blood donors during the COVID-19 outbreak in Milan. Blood Transfus. 2021;19(3):181-189.
4. Bruchez, A. et al. MHC class II transactivator CIITA induces cell resistance to Ebola virus and SARS-like coronaviruses.
5. Cui, J. et al. NLRC5 Negatively Regulates the NF-κB and Type I Interferon Signaling Pathways. Cell 141, 483–496.
6. Chang, K. T., Wu, H. J., Liu, C. W., Li, C. Y. & Lin, H. Y. A Novel Role of Arrhythmia-Related Gene KCNQ1 Revealed by Multi-Omic Analysis: Theragnostic Value and Potential Mechanisms in Lung Adenocarcinoma. Int. J. Mol. Sci. 23, (2022).
7. Rui, X., Shao, S., Wang, L. & Leng, J. Identification of recurrence marker associated with immune infiltration in prostate cancer with radical resection and build prognostic nomogram. BMC Cancer 19, (2019).
8. Gao, T. et al. Highly pathogenic coronavirus N protein aggravates inflammation by MASP-2-mediated lectin complement pathway overactivation. Signal Transduct. Target. Ther. 7, (2022).
9. Dai, Y. et al. Association of CXCR6 with COVID-19 severity: delineating the host genetic factors in transcriptomic regulation. Hum. Genet. 140, 1313–1328 (2021).
10. Mahesh, G. & Biswas, R. MicroRNA-155: A Master Regulator of Inflammation. J. Interferon Cytokine Res. 39, 321–330 (2019).

Disclaimer/Publisher's Note: The statements, opinions and data contained in all publications are solely those of the individual author(s) and contributor(s) and not of MDPI and/or the editor(s). MDPI and/or the editor(s) disclaim responsibility for any injury to people or property resulting from any ideas, methods, instructions or products referred to in the content.

https://doi.org/10.3390/ijms27020992
